# Anti-Poiseuille flow by spin Hall effect

**DOI:** 10.1093/pnasnexus/pgae547

**Published:** 2024-12-05

**Authors:** Junji Fujimoto, Wataru Koshibae, Sadamichi Maekawa

**Affiliations:** Department of Electrical Engineering, Electronics, and Applied Physics, Saitama University, Saitama 338-8570, Japan; RIKEN Center for Emergent Matter Science (CEMS), Wako, Saitama 351-0198, Japan; RIKEN Center for Emergent Matter Science (CEMS), Wako, Saitama 351-0198, Japan; Kavli Institute for Theoretical Sciences, University of Chinese Academy of Sciences, Beijing 100190, China; Advanced Science Research Center, Japan Atomic Energy Agency, Tokai 319-1195, Japan

**Keywords:** viscous electron fluid, spin Hall effect, charge and spin transports, magnetic skyrmion

## Abstract

Hydrodynamics is known to emerge in electron flow when the electron–electron interaction dominates over the other momentum-nonconserving scatterings. The hydrodynamic equation that describes the electric current includes viscosity, extending beyond the Ohmic flow. The laminar flow of such a viscous electron fluid in a sample with finite width is referred to as the Poiseuille flow, where the flow velocity is maximum at the center and decreases towards the edges of the sample. In this paper, we show a unique viscous electron fluid arising in electron systems exhibiting the spin Hall effect (spin Hall systems), where the charge and spin currents are coupled. Such a viscous electron fluid emerges even in noninteracting electron systems, and the current density exhibits a minimum at the center of a flow and a maximum at the edges, i.e. an anti-Poiseuille flow realizing. We also find that the spin accumulation by the spin Hall effect is connected to the electric current vorticity in two-dimensional (2D) spin Hall systems. Furthermore, we propose a novel guiding principle to manipulate topological magnetic textures from the hydrodynamic viewpoint. By solving the hydrodynamic equation in a 2D spin Hall system with a cavity and employing micromagnetic simulations for an attached chiral magnetic insulator, we demonstrate that spin accumulation near the cavity’s boundary leads to creating a magnetic skyrmion. Our research illuminates new aspects of electron hydrodynamics and spintronics, contributing significant insights to the fields.

**Significance Statement**

The laminar flow of fluids such as water and blood in narrow pipes forms the so-called Poiseuille flow, where the flow velocity is maximum at the center and decreases towards the edges. Such hydrodynamic behavior beyond Ohm’s law has recently been found in electron systems such as graphene. Here, we find a unique electron fluid that forms an anti-Poiseuille flow, where the current density is minimum at the center of the flow and maximum at the sample edges. This occurs in general electron systems exhibiting the spin Hall effect, where the charge and spin currents interact with each other. We propose a novel guiding principle to manipulate topological magnetic textures from the hydrodynamic viewpoint.

## Introduction

**Fig. 1.**
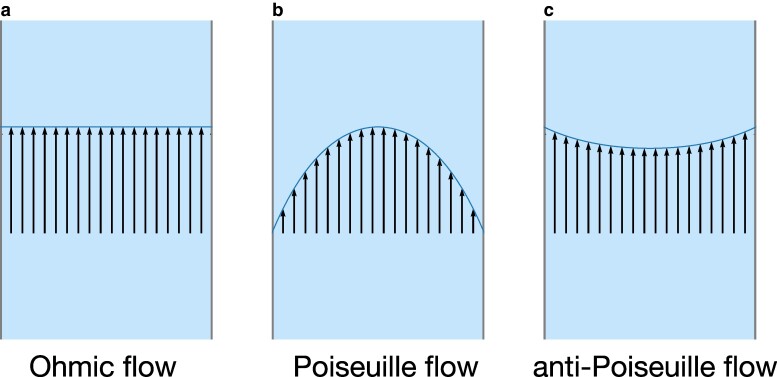
Various electric current profiles in a pipe flow. a) Ohmic flow is the simplest description of electric current in materials, and the electric current density is constant over the pipe. b) Poiseuille flow. Hydrodynamic nature is strongly appearing. We here consider the no-slip boundary condition. c) anti-Poiseuille flow. We show that the spin Hall system exhibits a more unique flow that the current density reaches a minimum at the center of the flow and a maximum at the edges.

Recent experiments show that electron transport is beyond Ohm’s law and becomes a viscous fluid in some materials with high purity, such as semiconductor systems (1–3), graphene (4–6), ${\text{Pd Co O}}_{2}$ (7), and ${\text{W Te}}_{2}$ (8, 9), which has given rise to the research field of electron hydrodynamics (10–12). Viscous electron fluid shows intriguing phenomena, such as negative resistance (4, 13) and vortices of electric current (3, 8, 13). The electric current vortices are characterized by nonzero vorticity $\omega_{e}=\nabla \times j_{e}$, where $j_{e}$ is the electric current density. It is important to emphasize that for the electric current described by Ohm’s law in the steady state, the vorticity is zero.^a^ Ohm’s law is given by $j_{e}^{\mathrm{Ohm}}=\sigma_{e}E$, where $\sigma_{e}$ is the electric conductivity of the material and E is the applied electric field, and hence the zero vorticity is proven as, $\omega_{e}^{\mathrm{Ohm}}=\nabla \times j_{e}^{\mathrm{Ohm}}=\sigma_{e}\nabla \times E=0$, considering that E=−∇ϕ with the electrostatic potential *ϕ*. Hence, the viscous electron fluid is distinct from the Ohmic transport as illustrated in Fig. 1a. The Poiseuille flow, depicted in Fig. 1b, is the typical evidence that the electric current becomes hydrodynamic since the flow slowing toward the edges stems from viscosity. As a more unique manifestation of electron hydrodynamics, an anomalous flow profile in charge-neutral graphene is predicted, named anti-Poiseuille flow (15), where the flow velocity exhibits a minimum at the center of a flow and a maximum at the edges (Fig. 1c). The anti-Poiseuille flow has attracted much attention recently (16–19). So far, the anti-Poiseuille flow has been discussed in specific materials in particular temperature regions, because the studies above are essentially based on electron–electron interaction.

In this paper, we demonstrate that electric current exhibits viscous fluid behavior in electron systems with the spin Hall effect (spin Hall systems), even in the absence of the electron–electron interaction. This is achieved by integrating out the spin degree of freedom. We show that the kinematic viscosity in the spin Hall systems is determined by the spin diffusion length and the transport lifetime. Furthermore, we reveal that the electron viscous fluid in spin Hall systems forms the anti-Poiseuille flow. Since the spin Hall effect is known to be one of the most general phenomena in spintronics, our finding that it induces the anti-Poiseuille flow of electrons represents a ubiquitous influence and opens a new avenue in the study of electron transport in metals and semiconductors. In two-dimensional (2D) spin Hall systems, the electric current vorticity is proportional to the spin accumulation due to the spin Hall effect. From the electron hydrodynamic perspective, we introduce a novel guiding principle for manipulating spin accumulation via geometric structures, aiming to induce topological transitions in magnetic textures. As a demonstration, we solve the Stokes equation and conduct micromagnetic simulations on a bilayer consisting of a specific spin Hall system and a chiral magnetic insulator (CMI) with a cavity structure. Our results reveal that spin accumulation near the cavity induces the formation of a magnetic skyrmion.

Here, we give a brief review of viscous electron fluid. Viscous electron fluid is described by the Stokes equation with the frictional force (20, 21):


(1) $$-\sigma_{e}\nabla \phi +l_{v}^{2}\nabla^{2}j_{e}-j_{e}=0,$$

where $l_{v}$ is the diffusion length of the vorticity, which is related to the kinematic viscosity *ν* and the transport lifetime *τ* through $l_{v}=\sqrt{\nu \tau }$. In the hydrodynamic viewpoint, the first term on the left-hand side in Eq. (1) represents the pressure, the second term represents viscosity, and the last term originates from the frictional force. When the viscosity is set to zero, ν=0, hence $l_{v}=0$, the Stokes Eq. (1) reduces into Ohm’s law. Hence, the Stokes equation is an extension of Ohm’s law. Next, one can find why $l_{v}$ is called the diffusion length of the vorticity by taking the rotation for the Stokes Eq. (1); the diffusion equation of the vorticity is obtained as $\nabla^{2}\omega_{e}=\omega_{e}/l_{v}^{2}$, where the typical diffusion length is given by $l_{v}$. Finally, noting that $\nabla \times \omega_{e}=-\nabla^{2}j_{e}$ because of the charge continuity equation in the steady state, $\nabla \cdot j_{e}=0$, we rewrite the Stokes Eq. (1) as


(2) $$j_{e}=-\sigma_{e}\nabla \phi -l_{v}^{2}\nabla \times \omega_{e}.$$

## Derivation of the hydrodynamic equation

Electron transport in systems exhibiting the direct and inverse spin Hall effects (22, 23), which we call spin Hall systems, can be regarded beyond Ohm’s law (see Supplementary material for the detailed explanation of the charge and spin transports). The inverse spin Hall effect induces an additional electric current density, $j_{e}^{\mathrm{SH}}=\theta_{\mathrm{SH}}\sum_{\alpha }\hat{\alpha }\times j_{s}^{\alpha }$ with α=x,y,z, where $j_{s}^{\alpha }$ is the spin current (in units of electric current density) whose spin polarization is directed to $\hat{\alpha }$ ($\hat{\alpha }$ is the *α*-directed unit vector), and $\theta_{\mathrm{SH}}$ is the spin Hall angle giving the spin-charge conversion efficiency. Hence, the total electric current density is given by


(3) $$j_{e}=j_{e}^{\mathrm{Ohm}}+j_{e}^{\mathrm{SH}}=\sigma_{e}E+\theta_{\mathrm{SH}}\sum_{\alpha }\hat{\alpha }\times j_{s}^{\alpha }.$$

Equation (3) indicates that electron transport in systems with the spin Hall effect is beyond Ohm’s law due to the contribution from the inverse spin Hall effect, $j_{e}^{\mathrm{SH}}$. We consider that the spin current $j_{s}^{\alpha }$ is given by


(4) $$j_{s}^{\alpha }=-\frac{\sigma_{e}}{e}\nabla \mu_{s}^{\alpha }+\theta_{\mathrm{SH}}\hat{\alpha }\times j_{e},$$

where $\mu_{s}^{\alpha }$ is the spin accumulation (in energy units) in the *α* direction with α=x,y,z and the spatially dependent $\mu_{s}$ leads to the spin density profile. The first term of Eq. (4) describes the diffusion spin current, and the second term is caused by the direct spin Hall effect. We here point out the similarity between the Stokes Eq. (2) and the electric current equation in the spin Hall systems. Inserting Eq. (4) into Eq. (3), we have


(5) $$j_{e}=-\sigma_{e}^{\prime }\nabla \phi +\theta_{\mathrm{SH}}\frac{\sigma_{e}^{\prime }}{e}\nabla \times \mu_{s},$$

where $\sigma_{e}^{\prime }$ is the renormalized electric conductivity^b^ given by


(6) $$\sigma_{e}^{\prime }=\frac{\sigma_{e}}{1+2\theta_{\mathrm{SH}}^{2}},$$

and $\mu_{s}=(\mu_{s}^{x},\mu_{s}^{y},\mu_{s}^{z})$.

In phenomenology, the spin accumulation in the steady state is distributed based on the spin diffusion equation,


(7) $$\nabla^{2}\mu_{s}^{\alpha }=\frac{\mu_{s}^{\alpha }}{\lambda_{s}^{2}},$$

where $\lambda_{s}$ is the spin diffusion length. By taking the rotation of Eq. (5) twice and using Eq. (7), then, we have $\lambda_{s}^{2}\nabla \times \omega_{e}=-(\theta_{\mathrm{SH}}\sigma_{e}^{\prime }/e)\nabla \times \mu_{s}$. Using this relation, we integrate out the spin accumulation from Eq. (5) so that we obtain


$$j_{e}=-\sigma_{e}^{\prime }\nabla \phi -\lambda_{s}^{2}\nabla \times \omega_{e},$$

or


(8) $$-\sigma_{e}^{\prime }\nabla \phi +\lambda_{s}^{2}\nabla^{2}j_{e}-j_{e}=0,$$

which is exactly the same as the Stokes equation in electron viscous fluid (20, 21). Eq. (8) is always held when the spin Hall effect arises. The diffusion length of the vorticity in the present case is given by the spin diffusion length. Eq. (8) is the first main result of this work, which indicates that the electric current in spin Hall systems behaves as an electron viscous fluid. The kinematic viscosity is given by the spin diffusion length and the transport lifetime as $\nu =\lambda_{s}^{2}/\tau$.

## Relation between electric current vorticity and spin accumulation

Next, we show that the electric current vorticity is directly connected to the spin accumulation in the 2D spin Hall systems. By taking the rotation of Eq. (5) and using Eq. (7), we obtain


(9) $$\omega_{e}=-\frac{\theta_{\mathrm{SH}}\sigma_{e}^{\prime }}{e\lambda_{s}^{2}}\mu_{s}+\frac{\theta_{\mathrm{SH}}\sigma_{e}^{\prime }}{e}\nabla (\nabla \cdot \mu_{s}).$$

Here, we consider a 2D system; $j_{e}$ flows in the *xy*-plane, so that the vorticity is in the *z*-direction as well as the spin accumulation due to the spin Hall effect $\mu_{s}=\mu_{s}^{z}\hat{z}$, which leads to $\nabla \cdot \mu_{s}=0$. Hence, the relation between the electric current vorticity and the spin accumulation is realized in the 2D spin Hall systems;


(10) $$\omega_{e}^{2D}=-\frac{\theta_{\mathrm{SH}}\sigma_{e}^{\prime }}{e\lambda_{s}^{2}}\mu_{s}^{z}\hat{z},$$

which shows that the electric current vorticity is directly connected to the spin accumulation.

In electron hydrodynamics, the observation of electric current vortices is crucial because these vortices originate from viscosity, as discussed in the Introduction. On the other hand, the spin accumulation has been directly observed through the optical Kerr effect (25–27), by the circular polarization of light (28), and by X-ray (29) and indirectly via the Hanle magnetoresistance (30–32) and the spin Hall magnetoresistance (33, 34). Following Eq. (10), we can say that the viscosity in spin Hall systems has been experimentally detected. Direct observations of electric current vortices in spin Hall systems through electrical methods, like nonlocal resistance measurements (4, 8) and the NV center method (5), are also highly desired.

The charge and spin transports in the spin Hall systems are connected to each other since the direct and the inverse spin Hall effects arise simultaneously. The spin accumulation at the boundaries is generated by the direct spin Hall effect, which is proportional to the spin Hall angle. The generated spin accumulation affects again the charge transport as the vorticity via the inverse spin Hall effect. Hence, the vorticity is proportional to $\theta_{\mathrm{SH}}^{2}$, whereas the spin accumulation is of the order of $\theta_{\mathrm{SH}}$, and the spin accumulation and the vorticity are connected via Eq. (10). The connection allows us to access the spin transport in the 2D spin Hall systems by solving the Stokes equation and taking the rotation of the electric current density.

The Stokes Eq. (8) together with the relation (10) provides the self-consistent treatment of charge and spin transports in the spin Hall systems with complex geometrical structures. Our approach fully incorporates the contributions of the spin Hall effect, particularly for large $\theta_{\mathrm{SH}}$ materials that have been developed recently (35), where the first order in $\theta_{\mathrm{SH}}$ theory does not apply. It is worth noting that numerical solvers for the Stokes equation are well established in the field of hydrodynamics, providing a robust framework for our analysis.

## Anti-Poiseuille flow

**Fig. 2.**
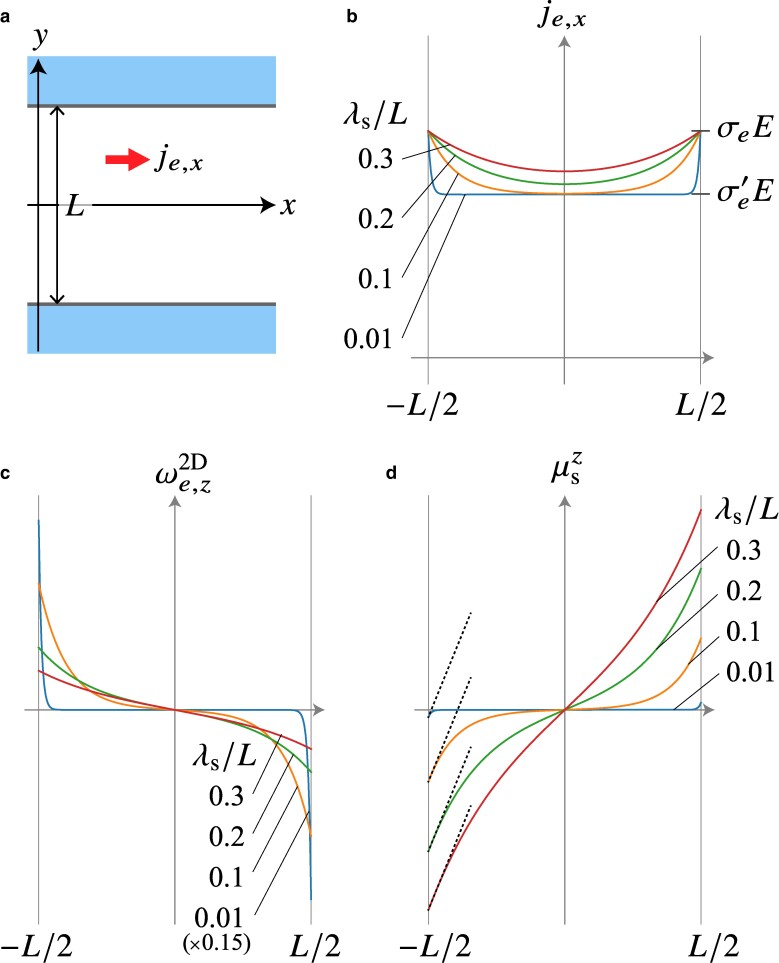
A typical example of a 2D pipe flow for the spin Hall systems. a) Geometrical setup of the pipe flow. The pipe width is given by *L*; the outside of the pipe is supposed to be vacuum. The hydrodynamic viscous flow along the pipe-length (*x*-) direction has a spatial distribution depending on the pipe-width (*y*-) direction due to the viscosity. b) Electric current density distribution in the spin Hall system for various spin diffusion length $\lambda_{s}$. The electric current density increases near the boundary. c) Electric current vorticity and d) corresponding spin accumulation for various spin diffusion lengths. The vorticity and spin accumulation are connected via Eq. (10). The magnitude of vorticity is larger near the boundary as the spin diffusion length is shorter, whereas the magnitude of spin accumulation is smaller. This behavior is discussed in Supplementary material.

The behavior near a boundary of the viscous electron fluid in the spin Hall systems is different from that of the viscous electron fluid caused by the electron–electron interaction for the boundary between the electron systems and the vacuum. The flow velocity of the conventional viscous electron fluid decreases near the boundary, based on the physical insight of viscosity. The reduction near the boundary is sometimes expressed by the slip length (36): the finite slip length indicates the corresponding decrease of the electric current density, and the infinite slip length means zero reduction. More importantly, the electric current density is considered to decrease near the boundary and the slip length would not be negative. However, the electric current density *increases* toward the boundary in the spin Hall systems, since the boundary condition originates from the fact that the spin current across the boundary should be zero;


(11) $$j_{s,⊥}^{\alpha }=\hat{n}\cdot j_{s}^{\alpha }=0,$$

for α=x,y,z with $\hat{n}$ being the unit vector perpendicular to the boundary yields


(12) $$j_{e,∥}=\sigma_{e}E_{∥}$$

at the boundary, where $j_{e,∥}=\hat{n}\times (j_{e}\times \hat{n})$ and $E_{∥}=\hat{n}\times (E\times \hat{n})$. We note that the zero electric current density at the boundary, i.e. the no-slip boundary condition, causes an unphysical result (see Supplementary material). Since the bare electric conductivity is larger than the renormalized one; $\sigma_{e}>\sigma_{e}^{\prime }$ (see Eq. (6)), we conclude that the electric current density increases as getting close to the boundary in the spin Hall systems; anti-Poiseuille flow arising. Its physical meaning is simple; the spin accumulation is induced by the direct spin Hall effect near the boundary, and the diffusion spin current of the accumulation is converted to the additional electric current due to the inverse spin Hall effect, so that the electric current density increases near the boundary. To understand the difference of the behaviors near the boundary of the spin Hall systems from the conventional viscous electron fluid, we consider a typical example of the 2D pipe flow (Fig. 2a). Solving the Stokes Eq. (8) for the geometrical configuration of 2D pipe flow, the general solution is given by $j_{e,x}(y)=Ae^{y/\lambda_{s}}+Be^{-y/\lambda_{s}}+\sigma_{e}^{\prime }E_{x}$, where $E_{x}=-\partial \phi /\partial x=\mathrm{const}$. is the applied electric field, and *A* and *B* are the unknown parameters that should be determined by the boundary conditions. The boundary conditions are given by $j_{e,x}(y=\pm L/2)=\sigma_{e}E_{x}$, so that we obtain $j_{e,x}(y)=[1+2\theta_{\mathrm{SH}}^{2}\text{cosh}(y/\lambda_{s})/\text{cosh}(L/2\lambda_{s})]\sigma_{e}^{\prime }E_{x}$. Figure 2b shows the electric current density distribution for the 2D spin Hall system.

Here, we show that we can discuss the spin transport based on the electric current vorticity through Eq. (10). For the 2D pipe flow in the spin Hall system, the vorticity is obtained as $\omega_{e,z}^{\mathrm{pipe}}=$  $-(2\theta_{\mathrm{SH}}^{2}\sigma_{e}^{\prime }E_{x}/\lambda_{s})\text{sinh}(y/\lambda_{s})/\text{cosh}(L/2\lambda_{s})$, which is shown in Fig. 2c. Inserting this expression into Eq. (10), the corresponding spin accumulation is given as $\mu_{s}^{\mathrm{pipe},z}=2e\lambda_{s}\theta_{\mathrm{SH}}E_{x}\text{sinh}(y/\lambda_{s})$  $/\text{cosh}(L/2\lambda_{s})$, which is plotted in Fig. 2d. Again, we note the important point that the vorticity is proportional to $\theta_{\mathrm{SH}}^{2}$ and that the spin accumulation is proportional to $\theta_{\mathrm{SH}}$. The spin accumulation is induced by the direct spin Hall effect and hence depends linearly on the spin Hall angle, while the electric current vorticity arises from the direct and inverse spin Hall effects, which leads to the dependence of $\theta_{\mathrm{SH}}^{2}$. As in Fig. 2d, the spin accumulation takes the smaller value as the spin diffusion length becomes shorter for the same spin Hall angle, which is reasonable since the shorter spin diffusion length leads to more rapid decay of the spin accumulation.

## Guiding principle of manipulating the spin accumulation by geometrical structures

Once given the hydrodynamic aspect of the spin Hall systems, the wealth of established knowledge of hydrodynamics brings a novel perspective on controlling spin accumulation. For example, hydrodynamics commonly treats systems with geometrical structures, such as a cavity and a notch, and the flow near the structures is accompanied by nonzero vorticity for the viscous fluid. Since vorticity is connected to spin accumulation in the spin Hall system, we propose a guiding principle for manipulating spin accumulation by geometrical structures. As a demonstration to utilize the designed spin accumulation, we numerically show a magnetic skyrmion creation via the electric current vorticity by solving the Stokes Eq. (8) with a cavity in a specific 2D spin Hall system combined with the micromagnetic simulation on a CMI attached to the spin Hall system.

## Solution of Stokes equation

**Fig. 3.**
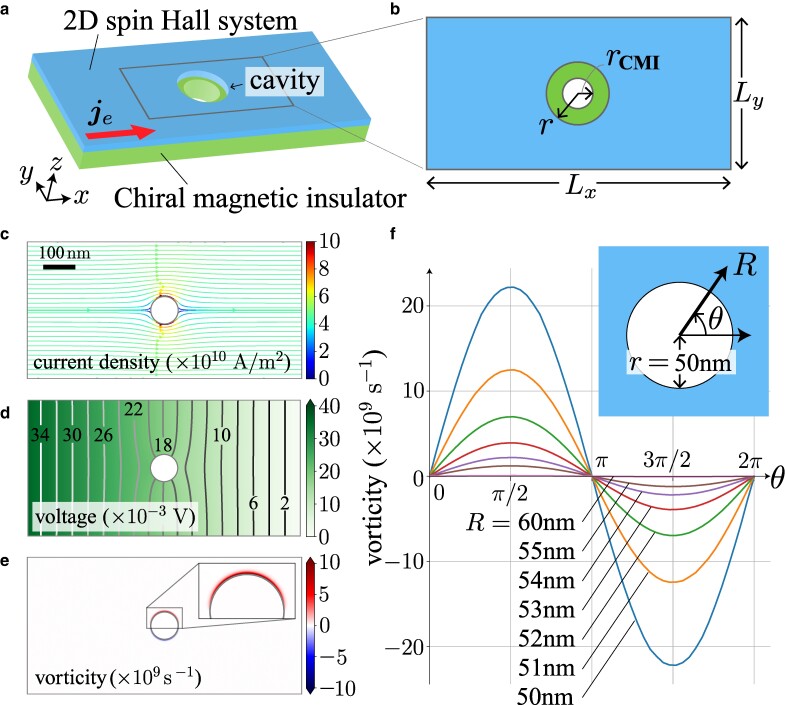
Numerical simulations of the Stokes equation. a) Schematics of the simulated system of the bilayer composed of 2D spin Hall system and CMI with cavity structure. b) Geometrical configuration of the simulations. The radius of the cavity in the spin Hall system is larger than that of CMI. c)–e) Resultant profiles of (c) the electric current density, (d) the voltage, and (e) the vorticity, solved based on the finite-element method (FEM). f) Angular dependence of the electric current vorticity near the cavity for various distances from the center of the cavity. The magnitude of the vorticity decays rapidly as *R* increases. The inset of (f) depicts the geometric definition of the distance *R* and the angle *θ*.

As shown in Fig. 3a, we consider a bilayer composed of a 2D spin Hall system and a CMI with cavity structure. We assume that the radius of the cavity in the CMI given by $r_{\mathrm{CMI}}$ is smaller than that in the spin Hall system denoted by *r*; $r>r_{\mathrm{CMI}}$, as depicted by Fig. 3b. We solve the Stokes Eq. (8) combined with the charge conservation $\nabla \cdot j_{e}=0$ with the boundary condition at cavity’s boundary, based on the FEM. The material parameters of ${\mathrm{Pt}}_{0.6}(\mathrm{MgO}{)}_{0.4}$ (35) are used as the spin Hall system;^c^ the resistivity is $\rho_{e}=1/\sigma_{e}^{\prime }=74\;\mu \Omega \cdot \;\mathrm{cm}$, the spin Hall angle is $\theta_{\mathrm{SH}}∼0.73$, and the spin diffusion length is $\lambda_{s}=1.75\;\mathrm{nm}$. See Supplementary material for more information on the FEM calculation. Note that the Stokes Eq. (8) with $\nabla \cdot j_{e}=0$ determines $j_{e}$ and *ϕ*. The solution of *ϕ* is consistent with the Laplace equation $\nabla^{2}\phi =0$. We have calculated the solutions within the linear response regime.

The FEM results are shown in Fig. 3c–e. Figure 3c depicts the flow profile of the electric current density $j_{e}$, and Fig. 3d shows the spatial profile of the scaler potential *ϕ*. The scaler potential monotonically decreases from the left side to the right side, but the electric current density has a complex structure near the cavity, which is related to the electric current vorticity $\omega_{e}=\omega_{e}\hat{z}$ shown by Fig. 3e. The electric current vorticity $\omega_{e}$ is plotted after converted into the vorticity in the frequency units; $\omega =\omega_{e}/en_{\mathrm{Pt}}$, where $n_{\mathrm{Pt}}=1.6\times 10^{28}/m^{3}$ is the electron density of pure Pt (39) (we assumed that the electron density of ${\mathrm{Pt}}_{0.6}(\mathrm{MgO}{)}_{0.4}$ is not so different from that of pure Pt). The inset of Fig. 3e is the close-up profile of the vorticity near the upper cavity. Since the radius of the cavity in the spin Hall system is set as r=50nm, which is much larger than the spin diffusion length $\lambda_{s}=1.75\;\mathrm{nm}$, the substantial vorticity occurs near by the cavity (Fig. 3f). The vorticity in the upper and lower sides of the cavity takes the positive and negative values, respectively, which is comprehended as the spin accumulation due to the direct spin Hall effect in the conventional viewpoint.

## Micromagnetic simulation

**Fig. 4.**
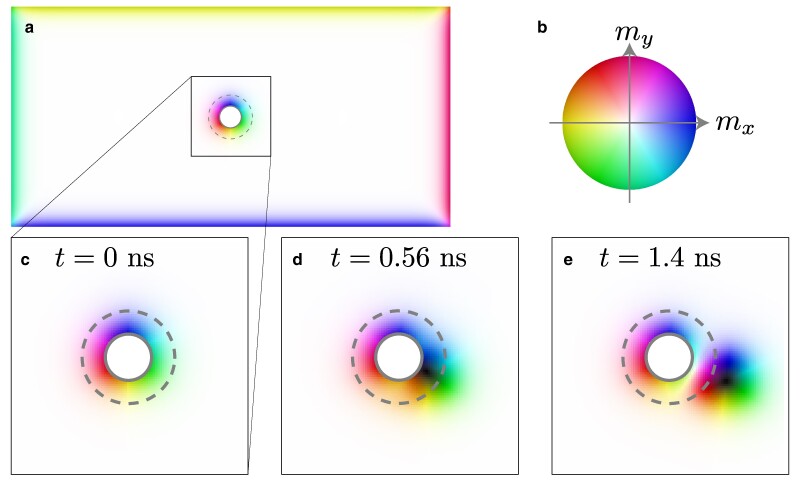
Micromagnetic simulation result. a) Snapshot of the system at t=0ns. b) Colormap of the magnetization. The color code specifies the in-plane component of the magnetic moment, e.g. the blue (yellow) means the positive (negative) $m_{x}$ direction. In the representation of the magnetic texture, the brightness represents the out-of-plane magnetic moment; that is, the white (black) is for $m_{z}=+1$  (−1). c)–e) Snapshots near the cavity at (c) t=0ns, (d) t=0.56ns, and (e) t=1.4ns by using the FEM result of the vorticity as the input. The successful magnetic skyrmion creation is observed. We set r=50nm and $r_{\mathrm{CMI}}=25\;\mathrm{nm}$.

Figure 4 shows the snapshots of the micromagnetic simulation result, which indicates the successful creation of a magnetic skyrmion. We compute the Landau–Lifshitz–Gilbert equation numerically by using the FEM result of the vorticity as the input, where we have presumed that the conduction electron spin in the spin Hall system is coupled to the magnetization in CMI through the *sd*-type exchange interaction, $H_{sd}=J_{sd}s\cdot m$, where m is the unit vector of the magnetization in CMI, $s=D\mu_{s}^{z}\hat{z}$ is the spin density with the density of state D, and $J_{sd}$ is the strength of the interaction. A candidate material for the CMI is ${\mathrm{Cu}}_{2}O{\mathrm{SeO}}_{3}$ (40), and we use the $D_{\mathrm{Pt}}∼3.3\times 10^{28}/\mathrm{eV}\;m^{3}$ (41) of pure Pt as D.

The detailed description of the micromagnetic simulation is given in Supplementary material.

We find that the magnetic skyrmion is successfully created, and the creation is accomplished by the effective magnetic field due to the spin accumulation near the boundary of the cavity through the *sd*-type exchange interaction. The locally induced strong effective field creates the magnetic skyrmion (42). This mechanism of skyrmion creation is essentially different from the conventional mechanisms based on the spin-transfer torque (43–45), and the spin–orbit torque (46). Note that we apply the external magnetic field perpendicular to the layers (the *z*-direction) to stabilize the magnetic skyrmion, but that is not essential for the skyrmion creation.

## Discussion

We now discuss the works on the unique flow profile that has nonmonotonic behaviors (16–19), especially on the anti-Poiseuille flow firstly proposed in Ref. (15). Such unique flows were predicted in some materials, such as charge-neutral graphene for particular temperature regions. Here, we have shown that the anti-Poiseuille flow can be found ubiquitously in the spin Hall systems since the spin Hall effect is experimentally observed in various materials at room temperature (23).

Here, we note the effect of friction from boundaries or the viscosity in the ordinary sense. In this paper, it has been proven that the effect of spin accumulation at the edges of a spin Hall system corresponds to the edge velocity distribution accompanied by the anti-Poiseuille flow. On the other hand, it is known that the effect of boundary friction influences the Poiseuille flow observed in conventional hydrodynamics. How the effect of boundary friction in a spin Hall system affects the spatial distribution of anti-Poiseuille flow, and thereby quantitatively modifies the analysis of spin accumulation, remains a subject for future study.

In Ref. (47), hydrodynamics in spin Hall systems was discussed by assuming the conventional hydrodynamic flow; electric current density decreases as going to the boundary. This conventional hydrodynamic flow is realized by the friction force by the boundary or simply the viscosity. However, we have obtained the anti-Poiseuille flow in spin Hall systems, which does not contain such viscosity in the conventional sense and reveals an essential difference beyond previous literature of Ref. (47).

Here, we discuss the comparison to our previous works on the connection between spin and vorticity (48–51). Our previous works on the skyrmion creation (48, 49) are based on the spin–vorticity coupling (52), which originates from general relativity. The present work has proposed an alternative mechanism of skyrmion creation, which is based on the spin–orbit coupling that comes from special relativity. Both mechanisms should be unified, which is one of the future works. It will be interesting to discuss the current vortex generation (50, 51) from the hydrodynamic viewpoint.

We here mention that the spin Hall systems have no Hall viscosity (53–55). The Hall viscosity is known to arise in the systems without time-reversal symmetry. However, we consider that the system has time-reversal symmetry, which leads to no Hall viscosity.

We also note the works on curvature-induced geometrical effects (56–60). It is known that curvature-induced geometical effects cause the enhancement of the coupling between spin and orbital motion. Here, we propose a guiding principle of spin manipulation by geometrical structures that is based on the hydrodynamic viewpoint, which has never been considered in the literature.

## Summary

In summary, we have shown that the electron transport in the spin Hall systems is described by the Stokes equation as in electron hydrodynamics. The kinematic viscosity is determined by the spin diffusion length and the transport lifetime. We discuss the boundary condition between the spin Hall system and the vacuum and then show that, unlike the viscous electron fluid that stems from the electron–electron interaction, the viscous electron fluid of the spin Hall systems forms the anti-Poiseuille flow in the pipe, where the current density is minimum at the center and maximum at the edges. In addition, the electric current vorticity is proportional to the spin accumulation due to the spin Hall effect in the 2D spin Hall systems. From the hydrodynamic viewpoint, we have proposed a novel guiding principle to manipulate topological magnetic textures. As the demonstration, we show the magnetic skyrmion creation in the bilayer system, which consists of the spin Hall system and the CMI with cavity structure. We hope that our theory and demonstration will stimulate both researchers in electron hydrodynamics and spintronics.

## Supplementary Material

pgae547_Supplementary_Data

## Data Availability

All study data are included in the article and/or Supplementary material.

## Notes

a In the cases where the electric conductivity is varying spatially, such as in gradient materials (14), the electric current vorticity is finite even when Ohm’s law is realized, but the viscosity in such systems is zero.

b Sometimes Eq. (5) in which $\sigma_{e}^{\prime }$ is replaced with $\sigma_{e}$ is used as a starting point in phenomenological treatments (24). In Supplementary material, we discuss the relation between Eqs. (3) and (5).

c Recently, alloys with large spin Hall angle ($\theta_{\mathrm{SH}}≳0.35$) have been reported, such as $\theta_{\mathrm{SH}}≃0.35$ for ${\mathrm{Cu}}_{1-x}{\mathrm{Tb}}_{x}$ with x=0.61 (37), $\theta_{\mathrm{SH}}≃0.6$ for ${\mathrm{Pd}}_{1-x}{\mathrm{Pt}}_{x}$ with x=0.75 (38), and $\theta_{\mathrm{SH}}≃0.73$ for ${\mathrm{Pt}}_{1-x}{\left(\mathrm{MgO}\right)}_{x}$ with x=0.4 (35). For these alloys, $\theta_{\mathrm{SH}}^{2}≳0.12$.

## Preprints

This manuscript was posted on a preprint: https://doi.org/10.48550/arXiv.2303.17234.
