# Supplementary material for: Anti-Poiseuille flow by spin Hall effect
Source: PNAS Nexus. 2024 Dec 5;3(12):pgae547. doi: 10.1093/pnasnexus/pgae547 (PMC11646127; doi:10.1093/pnasnexus/pgae547)
Supplement: pgae547_Supplementary_Data [file pgae547_supplementary_data.zip › PNASNEXUS-PNASNEXUS-2024-00256-TRRR-s01.pdf]

Supplementary Material:
Anti-Poiseuille flow by spin Hall effect

Junji Fujimoto,[1] Wataru Koshibae,[2] and Sadamichi Maekawa[2, 3, 4]

[1] *Department of Electrical Engineering, Electronics, and Applied Physics, Saitama University, Saitama, 338-8570, Japan**
[2] *RIKEN Center for Emergent Matter Science (CEMS), Wako, Saitama 351-0198, Japan*
[3] *Kavli Institute for Theoretical Sciences, University of Chinese Academy of Sciences, Beijing, 100190, China*
[4] *Advanced Science Research Center, Japan Atomic Energy Agency, Tokai 319-1195, Japan*
(Dated: November 28, 2024)

CONTENTS

I. Relation between the two starting points about the spin Hall effect 1
II. Relation to previous studies 3
III. Discussion on boundary conditions 3
IV. Vorticity profile in pipe flow and Stokes' theorem 4
V. Spin accumulation profile at boundary 4
VI. Note on spin Hall angle $\theta_{\rm SH}$ and spin diffusion length $\lambda_{\rm s}$ 5
VII. Details of the FEM calculation 5
VIII. Details of the micromagnetic simulation 6
IX. Electric field distribution 7
References 7

I. RELATION BETWEEN THE TWO STARTING POINTS ABOUT THE SPIN HALL EFFECT

There are two starting points to discuss phenomena related to the direct and inverse spin Hall effects [1–8]. In the first starting point, the inverse spin Hall current $\boldsymbol{j}_e^{\rm SH}$ and the direct spin Hall current $\boldsymbol{j}_{\rm s}^{{\rm SH},\alpha}$ are given by

$$\boldsymbol{j}_e^{\rm SH} = \theta_{\rm SH} \sum_{\alpha=x,y,z} \hat{\alpha} \times \boldsymbol{j}_{\rm s}^{\alpha}, \tag{S1.1}$$

$$\boldsymbol{j}_{\rm s}^{{\rm SH},\alpha} = \theta_{\rm SH}\hat{\alpha} \times \boldsymbol{j}_e, \tag{S1.2}$$

where $\boldsymbol{j}_e$ is the (total) electric current, $\boldsymbol{j}_{\rm s}^{\alpha}$ is the (total) spin current whose spin polarization is directed to $\hat{\alpha}$ with $\alpha = x, y, z$, $\theta_{\rm SH}$ is the spin Hall angle describing the conversion efficiency, and $\hat{\alpha}$ is the unit vector of the α direction. The complete formulas of the electric current and spin current are obtained as

$$\boldsymbol{j}_e = \boldsymbol{j}_e^{\rm Ohm} + \boldsymbol{j}_e^{\rm SH} = \sigma_e \boldsymbol{E} + \theta_{\rm SH} \sum_{\alpha=x,y,z} \hat{\alpha} \times \boldsymbol{j}_{\rm s}^{\alpha}, \tag{S1.3}$$

$$\boldsymbol{j}_{\rm s}^{\alpha} = \boldsymbol{j}_{\rm s}^{{\rm diffusion},\alpha} + \boldsymbol{j}_{\rm s}^{{\rm SH},\alpha} = -\frac{\sigma_e}{e}\boldsymbol{\nabla}\mu_{\rm s}^{\alpha} + \theta_{\rm SH}\hat{\alpha} \times \boldsymbol{j}_e, \tag{S1.4}$$

where $\boldsymbol{j}_e^{\rm Ohm} = \sigma_e \boldsymbol{E}$ is the Ohmic current with the conductivity σ_e, and $\boldsymbol{j}_{\rm s}^{{\rm diffusion},\alpha} = -(\sigma_e/e)\boldsymbol{\nabla}\mu_{\rm s}^{\alpha}$ is the diffusion spin current with the spin accumulation $\mu_{\rm s}^{\alpha}$.

* E-mail address: jfujimoto@mail.saitama-u.ac.jp

In the second starting point, the inverse and direct spin Hall currents are given as

$$\tilde{\boldsymbol{j}}_e^{\rm SH} = -\theta_{\rm SH}\frac{\tilde{\sigma}_e}{e}\sum_{\alpha=x,y,z}\hat{\alpha}\times\boldsymbol{\nabla}\mu_{\rm s}^{\alpha}, \tag{S1.5}$$

$$\tilde{\boldsymbol{j}}_{\rm s}^{{\rm SH},\alpha} = \theta_{\rm SH}\tilde{\sigma}_e\hat{\alpha}\times\boldsymbol{E}, \tag{S1.6}$$

where we have expressed the inverse spin Hall current by $\tilde{\boldsymbol{j}}_e^{\rm SH}$ and the direct spin Hall current $\tilde{\boldsymbol{j}}_{\rm s}^{{\rm SH},\alpha}$ with the tilde symbols to distinguish Eqs. (S1.1) and (S1.2), respectively. The conductivity in this starting point is denoted by $\tilde{\sigma}_e$. We note that the following relation is important for our theory,

$$\sum_{\alpha=x,y,z}\hat{\alpha}\times\boldsymbol{\nabla}\mu_{\rm s}^{\alpha} = -\boldsymbol{\nabla}\times\boldsymbol{\mu}_{\rm s}. \tag{S1.7}$$

For these definitions, the total electric and spin currents are given by

$$\tilde{\boldsymbol{j}}_e = \tilde{\boldsymbol{j}}_e^{\rm Ohm} + \tilde{\boldsymbol{j}}_e^{\rm SH} = \tilde{\sigma}_e\boldsymbol{E} - \theta_{\rm SH}\frac{\tilde{\sigma}_e}{e}\sum_{\alpha=x,y,z}\hat{\alpha}\times\boldsymbol{\nabla}\mu_{\rm s}^{\alpha}, \tag{S1.8}$$

$$\tilde{\boldsymbol{j}}_{\rm s}^{\alpha} = \tilde{\boldsymbol{j}}_{\rm s}^{{\rm diffusion},\alpha} + \tilde{\boldsymbol{j}}_{\rm s}^{{\rm SH},\alpha} = -\frac{\tilde{\sigma}_e}{e}\boldsymbol{\nabla}\mu_{\rm s}^{\alpha} + \theta_{\rm SH}\tilde{\sigma}_e\hat{\alpha}\times\boldsymbol{E}. \tag{S1.9}$$

Equations (S1.8) and (S1.9) are regarded as the linear response formulas of the electric and spin currents to the forces $\boldsymbol{E}$ and $-\boldsymbol{\nabla}\mu_{\rm s}^{\alpha}$.

We show that Eqs. (S1.3) and (S1.4) are essentially equivalent to Eqs. (S1.8) and (S1.9) for the linear responses to the forces. Substituting Eq. (S1.4) into Eq. (S1.3), we find

$$\begin{aligned}\boldsymbol{j}_e &= \sigma_e\boldsymbol{E} + \theta_{\rm SH}\sum_{\alpha=x,y,z}\hat{\alpha}\times\left(-\frac{\sigma_e}{e}\boldsymbol{\nabla}\mu_{\rm s}^{\alpha} + \theta_{\rm SH}\hat{\alpha}\times\boldsymbol{j}_e\right)\\ &= \sigma_e\boldsymbol{E} - \theta_{\rm SH}\frac{\sigma_e}{e}\sum_{\alpha=x,y,z}\hat{\alpha}\times\boldsymbol{\nabla}\mu_{\rm s}^{\alpha} + \theta_{\rm SH}^2\sum_{\alpha=x,y,z}\hat{\alpha}\times(\hat{\alpha}\times\boldsymbol{j}_e).\end{aligned} \tag{S1.10}$$

From the vector calculus identity, $\hat{\alpha}\times(\hat{\alpha}\times\boldsymbol{j}_e) = \hat{\alpha}(\hat{\alpha}\cdot\boldsymbol{j}_e) - \boldsymbol{j}_e$, the last term of the right hand side reads

$$\sum_{\alpha=x,y,z}\hat{\alpha}\times(\hat{\alpha}\times\boldsymbol{j}_e) = \sum_{\alpha=x,y,z}\hat{\alpha}(\hat{\alpha}\cdot\boldsymbol{j}_e) - \sum_{\alpha=x,y,z}\boldsymbol{j}_e = \boldsymbol{j}_e - 3\boldsymbol{j}_e = -2\boldsymbol{j}_e, \tag{S1.11}$$

and hence we obtain

$$\boldsymbol{j}_e = \frac{\sigma_e}{1+2\theta_{\rm SH}^2}\boldsymbol{E} - \frac{\theta_{\rm SH}}{e}\frac{\sigma_e}{1+2\theta_{\rm SH}^2}\sum_{\alpha=x,y,z}\hat{\alpha}\times\boldsymbol{\nabla}\mu_{\rm s}^{\alpha}. \tag{S1.12}$$

By introducing the renormalized electric conductivity σ_e' as

$$\sigma_e' = \frac{\sigma_e}{1+2\theta_{\rm SH}^2} \tag{S1.13}$$

as in the main text, we get the same equation as Eq. (S1.8) except the difference between σ_e' and $\tilde{\sigma}_e$. Inserting Eq. (S1.12) into Eq. (S1.4), we have

$$\begin{aligned}\boldsymbol{j}_{\rm s}^{\alpha} &= -\frac{\sigma_e}{e}\boldsymbol{\nabla}\mu_{\rm s}^{\alpha} + \theta_{\rm SH}\hat{\alpha}\times\left(\sigma_e'\boldsymbol{E} - \theta_{\rm SH}\frac{\sigma_e'}{e}\sum_{\alpha=x,y,z}\hat{\alpha}\times\boldsymbol{\nabla}\mu_{\rm s}^{\alpha}\right)\\ &= -\frac{\sigma_e'}{e}\boldsymbol{\nabla}\mu_{\rm s}^{\alpha} + \theta_{\rm SH}\sigma_e'\hat{\alpha}\times\boldsymbol{E},\end{aligned} \tag{S1.14}$$

which is equivalent to Eq. (S1.9) except the difference between σ_e' and $\tilde{\sigma}_e$.

From the above calculations, we find that σ_e in the first phenomenology is understood as the conductivity that does not include the direct and inverse spin Hall effects, the renormalized conductivity σ_e' is the conductivity that contains the spin Hall effects, and $\tilde{\sigma}_e$ in the second phenomenology should be equivalent to σ_e' in the first one. The conductivity (or resistivity) which is observed in experiments corresponds to σ_e' and $\tilde{\sigma}_e$.

II. RELATION TO PREVIOUS STUDIES

Here, we comment on the previous works on the connection between spin and vorticity [9–18]. Based on the general relativistic theory, it was shown that the electron spin couples to the mechanical rotation, or vorticity in the local picture, through the spin-rotation (spin-vorticity) coupling [9, 12, 19], which was experimentally observed in liquid metals [10, 14, 15, 17] and by surface acoustic waves [11, 16]. An attempt to interpret the spin-orbit coupling as the spin-vorticity coupling was made based on microscopic calculations [13, 18]. In Ref. [18], the relation between spin density and electric current vorticity is discussed, where a specific electron viscosity is assumed, while we do not assume any electron viscosity and find that the electron viscosity is induced by the spin Hall effect in the present work.

III. DISCUSSION ON BOUNDARY CONDITIONS

Here, we discuss the boundary conditions between the spin Hall system and the vacuum. In conventional hydrodynamics, a slip length is sometimes introduced [20, 21] to describe the boundary conditions with the vacuum boundary, and can be evaluated from a microscopic model of boundary scattering [22]. However, spin Hall systems (as defined by Eqs. (3), (4), and (7) in the main text) cannot be described by the slip length, since the electric current density does not decrease near the boundary but rather increases due to the direct and inverse spin Hall effects. This leads to the realization of an anti-Poiseuille flow in generic spin Hall systems.

Below, we show that the no-slip boundary condition is not allowed in the spin Hall systems. Before showing that, we discuss the case of no-slip condition for standard electron hydrodynamics [23, 24]. In standard hydrodynamics, both the electrostatic potential (or pressure) and the vorticity contribute to the electric current.

$$\boldsymbol{j}_e = -\sigma_e \boldsymbol{\nabla}\phi - l_v^2 \boldsymbol{\nabla} \times \boldsymbol{\omega}_e, \tag{S3.1}$$

where the electrostatic potential and the vorticity are determined by the following equations,

$$\nabla^2 \phi = 0, \qquad \nabla^2 \boldsymbol{\omega}_e = \frac{\boldsymbol{\omega}_e}{l_v^2}. \tag{S3.2}$$

These two equations are obtained by taking the divergence and curl of the Stokes equation with the charge continuity equation, respectively. The no-slip condition, $j_{e,\parallel}\big|_{\text{boundary}} = 0$, can be realized when

$$j_{e,\parallel}\big|_{\text{boundary}} = -\sigma_e \hat{t} \cdot \boldsymbol{\nabla}\phi\big|_{\text{boundary}} - l_v^2 \hat{t} \cdot (\boldsymbol{\nabla} \times \boldsymbol{\omega}_e)\big|_{\text{boundary}} = 0, \tag{S3.3}$$

where $\hat{t}$ is a parallel unit vector to the boundary. Hence, ϕ and $\boldsymbol{\omega}_e$ are coupled with each other in standard electron hydrodynamics.

However, the 2D spin Hall systems have quite different behavior from standard hydrodynamics, as discussed below. In the present work, we reveal that the electric current vorticity is proportional to the spin accumulation in the 2D spin Hall systems. Therefore, the equation for the vorticity is equivalent to the spin diffusion equation,

$$\nabla^2 \mu_{\text{s}}^z = \frac{\mu_{\text{s}}^z}{\lambda_{\text{s}}^2}, \tag{S3.4}$$

where μ_{s}^z is the perpendicular component of the spin accumulation to the 2D spin Hall system, and λ_{s} is the spin diffusion length. We emphasize that the spin accumulation profile, and therefore the vorticity profile, is strongly restricted by the boundary conditions for the spin current, which is quite different from standard hydrodynamics. The spin current density perpendicular to the boundary is given by

$$j_{\text{s},\perp}^z = -\frac{\sigma_e}{e} \hat{n} \cdot \boldsymbol{\nabla}\mu_{\text{s}}^z + \theta_{\text{SH}} j_{e,\parallel}, \tag{S3.5}$$

where $\hat{n}$ is the normal unit vector to the boundary, $j_{\text{s},\perp}^z = \hat{n} \cdot \boldsymbol{j}_{\text{s}}^z$, and $j_{e,\parallel} = (\hat{n} \times \hat{z}) \cdot \boldsymbol{j}_e$.

If we take the no-slip boundary condition, $j_{e,\parallel}\big|_{\text{boundary}} = 0$, with no spin current across the boundary $j_{\text{s},\perp}^z\big|_{\text{boundary}} = 0$, we have

$$\hat{n} \cdot \boldsymbol{\nabla}\mu_{\text{s}}^z\Big|_{\text{boundary}} = 0. \tag{S3.6}$$

We here present an example that contradicts the no-slip condition: the typical pipe flow with the pipe width L (Fig. 2. (a) in the main text). The solution of the spin diffusion equation in the case is shown as

$$\mu_{\text{s}}^z(r) = A e^{y/\lambda_{\text{s}}} + B e^{-y/\lambda_{\text{s}}}, \tag{S3.7}$$

where A and B are the constants determined by the boundary conditions. In this case, the condition (S3.6) concludes $A = B = 0$, which leads to no spin accumulation, hence no vorticity.

The zero spin accumulation profile means no vorticity and the Ohmic current realizing:

$$\boldsymbol{j}_e = \sigma_e \boldsymbol{E}. \tag{S3.8}$$

We state that $E_\parallel|_{\rm boundary} = 0$ cannot be consistent with the Ohmic current. Hence, the no-slip boundary condition is not allowed in the spin Hall systems. We conclude that the condition (S3.6) results in a zero spin accumulation profile, which is unphysical in the spin Hall systems.

In order to reconcile the concept of slip length with spin Hall systems, it will be necessary to extend the underlying equation (3) in the main text to include the kinematic viscosity. For consideration, the spin scattering at a boundary may play a role. However, for commonly-used spintronic materials, the characteristic length determined by the kinematic viscosity is expected to be much smaller than the spin diffusion length; hence, Eq. (3) remains applicable, and our present theory is valid. Extending this theory to account for kinematic viscosity is a task for future work.

IV. VORTICITY PROFILE IN PIPE FLOW AND STOKES' THEOREM

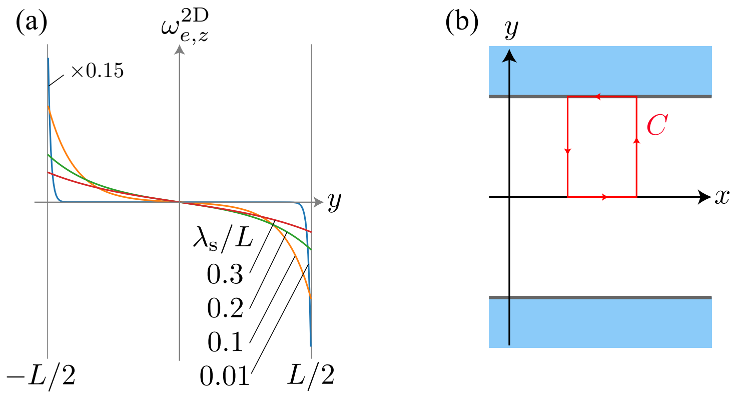

FIG. S1: (a) Spatial profile of vorticity for the pipe flow. (b) Closed path C for Stokes' theorem.

Figure S1 (a) depicts the vorticity profile same as in Fig. 2 in the main text. The larger vorticity is obtained near the boundary as the spin diffusion length is shorter. Seemingly, this behavior is odd but can be understood by Stokes' theorem; taking the closed path C as shown in Fig. S1 (b),

$$\oint_C \boldsymbol{j}_e \cdot {\rm d}\boldsymbol{l} = \iint_S (\boldsymbol{\nabla} \times \boldsymbol{j}_e) \cdot {\rm d}\boldsymbol{S}\,, \tag{S4.1}$$

where S is the area enclosed by the path C. This equation leads to

$$(\sigma_e - \sigma_e')E_x = -\int_0^{L/2} {\rm d}y\, \omega_{e,z}^{\rm pipe} \tag{S4.2}$$

for short spin diffusion length $\lambda_{\rm s}/L \ll 1$. This equation does not depend on the spin diffusion length $\lambda_{\rm s}$, while the integrand $\omega_{e,z}^{\rm pipe}$ depends on $\lambda_{\rm s}$. The area in which the vorticity contributes significantly to the integral is narrower as $\lambda_{\rm s}$ is shorter, so that the vorticity itself at the boundary should increase as shorter $\lambda_{\rm s}$ to match the consistency.

V. SPIN ACCUMULATION PROFILE AT BOUNDARY

We here comment on the values of the diffusion spin current at the boundaries $y = \pm L/2$ in the pipe flow. The gradient of the spin accumulation gives the diffusion spin current as

$$j_{{\rm s},y}^{{\rm diff},z} = -\frac{\sigma_e'}{e}\frac{\partial \mu_{\rm s}^{{\rm pipe},z}}{\partial y} \tag{S5.1}$$

and the diffusion spin current at the boundary $y = \pm L/2$ is independent of the value of the spin diffusion length λ_s, as plotted by the dashed lines near $y = -L/2$ in Fig. S2, which comes from the boundary condition that the total spin current should vanish at the boundary;

$$j_{s,y}^{\mathrm{diff},z}|_{y=\pm L/2} = \theta_{\mathrm{SH}}\sigma_e' E_x = -j_{s,y}^{\mathrm{SH},z}, \tag{S5.2}$$

where $j_{s,y}^{\mathrm{SH},z}$ is the spin current due to the direct spin Hall effect.

VI. NOTE ON SPIN HALL ANGLE θ_{SH} AND SPIN DIFFUSION LENGTH λ_s

We here note the spin Hall angle θ_{SH} and the spin diffusion length λ_s. Although both phenomenological parameters may depend on the same spin-orbit coupling strength, they depend on many other microscopic parameters. In particular, the spin diffusion length is given by $\lambda_s = \sqrt{D\tau_s}$, where D is the diffusion constant and τ_s is the spin relaxation time. Many mechanisms contribute to the spin relaxation time, such as the scatterings by magnetic impurities and spin-orbit impurities, and the intrinsic spin-orbit coupling (dephasing); $\tau_s^{-1} = \tau_{\mathrm{mag}}^{-1} + \tau_{\mathrm{soc}}^{-1} + \tau_{\mathrm{deph}}^{-1} + \cdots$. Hence, we consider the above two phenomenological parameters can be determined independently.

VII. DETAILS OF THE FEM CALCULATION

Here, we show the detail configuration of the finite element method (FEM) calculation shown in Fig. 3 of the main text. We compute the FEM calculation by using a free FEM software, FreeFEM++ [25]. Taking the length, time, voltage units as $l_0 = 1$ nm, $t_0 = 1$ ps, and $\phi_0 = 1\ \mu$V, respectively, we set the sample geometry as $L_x = 1000\ l_0$, $L_y = 500\ l_0$, and $r = 50\ l_0$ [Fig.S3 (a)]. We apply the electric voltage between the sample; $\varphi = 36$ mV $= 36000\ \phi_0$.

To obtain the boundary condition at the cavity edge, we first solve the Laplace equation for $\Phi = \phi/\phi_0$,

$$\tilde{\nabla}^2\Phi = 0 \tag{S7.1}$$

with the boundary conditions $\Phi|_{\mathrm{Boundary1}} = \varphi/\phi_0$, $\phi|_{\mathrm{Boundary3}} = 0$, and $\partial\Phi/\partial\boldsymbol{n}|_{\mathrm{Boundary2,4,5}} = 0$ for the sample geometry, where $\tilde{\boldsymbol{\nabla}} = l_0\boldsymbol{\nabla}$. Here $\boldsymbol{n}$ is the unit normal vector to the boundary. From the solution of Φ, we compute the electric current density at the boundaries; $\boldsymbol{J}|_{\mathrm{Boundary}} = -\tilde{\boldsymbol{\nabla}}\Phi|_{\mathrm{Boundary}}$, where $\boldsymbol{J} = \boldsymbol{j}_e/j_0$ with $j_0 = \sigma_e'\phi_0/l_0$. We take the FEM mesh as shown in Fig. S3 (b).

The time-dependent Stokes equation of the spin Hall systems is nondimensionalized as

$$\tilde{\tau}\frac{\partial \boldsymbol{J}}{\partial T} - \tilde{\boldsymbol{\nabla}}\Phi + \tilde{l}_s^2\tilde{\nabla}^2\boldsymbol{J} - \boldsymbol{J} = 0, \tag{S7.2}$$

where we have introduced the following variables,

$$T = t/t_0, \qquad \tilde{\tau} = \tau/t_0, \qquad \tilde{l}_s = l_s/l_0. \tag{S7.3}$$

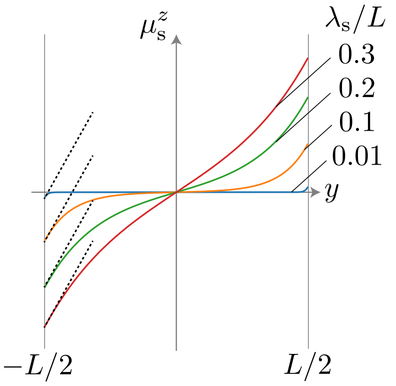

FIG. S2: Spin accumulation of the 2D pipe flow for various spin diffusion lengths (same as Fig. 2 in the main text). The gradient of the spin accumulation is proportional to the diffusion spin current, which takes the same value at the boundary $y = \pm L/2$ for different spin diffusion lengths with the same spin Hall angle, as plotted by the dashed line near $y = -L/2$.

The time step is set $dt = 0.014\, t_0$.

We set the boundary conditions for Boundary 1, 2 and 4 [see Fig. S3 (a)] as $\boldsymbol{J}|_{\text{Boundary1,2,4}} = -f_{\text{on}}(T)\tilde{\boldsymbol{\nabla}}\Phi|_{\text{Boundary1,2,4}}$, and for Boundary 3 as $\Phi|_{\text{Boundary3}} = 0$, where $f_{\text{on}}(T)$ is the switch-on function;

$$f_{\text{on}}(T) = 1 - e^{-(T-T_0)/T_{\text{on}}} \tag{S7.4}$$

with $T_0 = 0.5$ ps and $T_{\text{on}} = 0.1$ ps [1]. The boundary condition for Boundary 5 is given as $\boldsymbol{J}_{\text{Boundary5}} = -(1+2\theta_{\text{SH}}^2)f_{\text{on}}(T)\tilde{\boldsymbol{\nabla}}\Phi|_{\text{Boundary5}}$, which originates from the vanishment of the spin current across the boundary. Note that T_{on} can be seen much longer than dt; $T_{\text{on}} \gg dt$, which allows us the quasistatic approximation.

We introduce the unit of vorticity $\omega_0 = j_0/l_0$ and the nondimensionalized quantity;

$$\boldsymbol{\Omega} = \omega_e/\omega_0. \tag{S7.5}$$

The simulation results are shown in Fig. 3 in the main text.

Then, the spin accumulation μ_s^z is expressed as

$$\mu_s^z = -\frac{e\phi_0 l_s^2}{\theta_{\text{SH}}}\boldsymbol{\Omega}\cdot\hat{z}. \tag{S7.6}$$

We estimate the spin density $\boldsymbol{s} = s\hat{z}$ from μ_s^z by multiplying the density of state $\mathscr{D} \sim \mathscr{D}_{\text{Pt}} \sim 3.3 \times 10^{28}$ /eV m^3 [26],

$$s = \mathscr{D}\mu_s^z, \tag{S7.7}$$

and obtain the spin number in the unit volume of the discretized system v as

$$s_{\boldsymbol{r}} = vs, \tag{S7.8}$$

which is used as the input of the micromagnetic simulation in the following section, where we set $v = a^2 d$ with the discretized length a (see the following section) and the thickness of the spin Hall system $d = 10$ nm.

VIII. DETAILS OF THE MICROMAGNETIC SIMULATION

To study the dynamics of the spatially slowly-varying magnetic-texture, we discretize the system with a unit length $a = 25$ Å, i.e., the normalized magnetic moments $\boldsymbol{m_r}$ are on the lattice point of the two-dimensional square lattice with a system size 401×201.

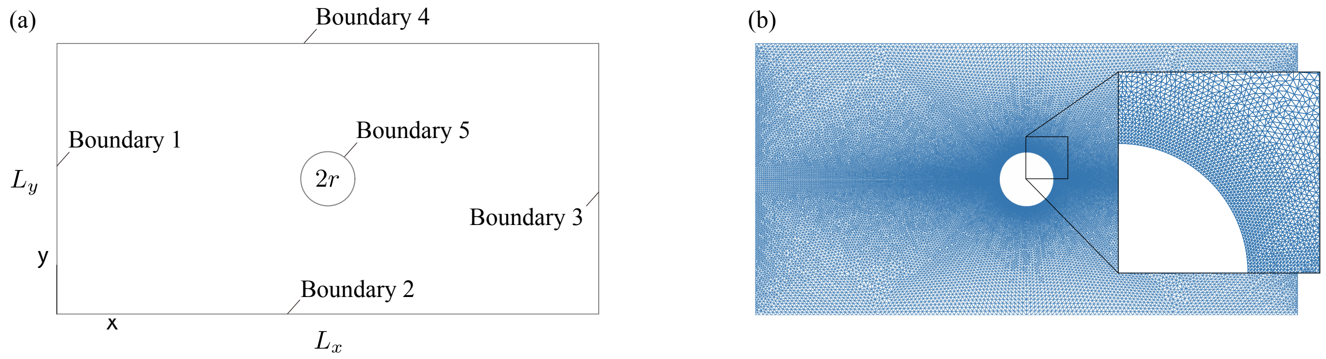

FIG. S3: (a) Geometrical configuration and boundary indexes. (b) FEM mesh.

[1] The value of T_{on} is estimated as $T_{\text{on}} \sim L/R$, where $L \sim \mu_0 l/2\pi$ is the inductance and $R = \rho l/wd$ is the resistance with the system width $w \sim 5$ μm and thickness $d \sim 10$ nm and with the vacuum permeability $\mu_0 = 4\pi \times 10^{-7}$ H/m. Assuming $\rho \sim 10^{-7}$ Ω m, which leads to $T_{\text{on}} \simeq 0.1$ ps.

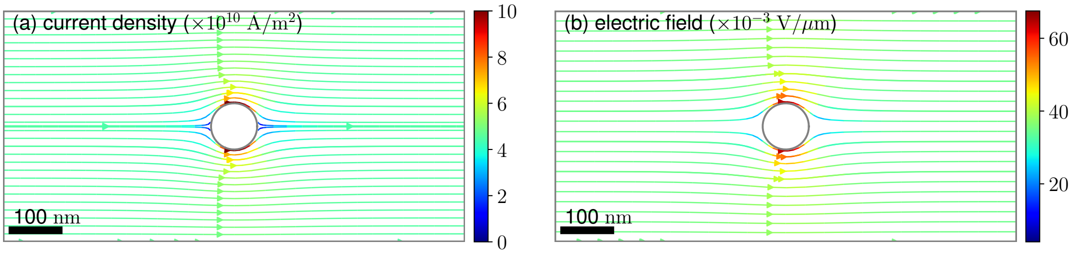

FIG. S4: (a) Electric current profile and (b) electric field profile obtained from the FEM calculation.

The model Hamiltonian in the chiral magnetic insulator (CMI) is expressed by $H = H_0 + H_{sd}$,

$$H_0 = -J \sum_{\langle \boldsymbol{r},\boldsymbol{r}'\rangle} \boldsymbol{m_r} \cdot \boldsymbol{m_{r'}} + D_{\text{bulk}} \sum_{e=x,y} \sum_{\boldsymbol{r}} (\boldsymbol{m_r} \times \boldsymbol{m}_{\boldsymbol{r}+\hat{e}}) \cdot \hat{e}$$
$$+ D_{\text{int}} \sum_{\boldsymbol{r}} \left[(\boldsymbol{m_r} \times \boldsymbol{m}_{\boldsymbol{r}+\hat{x}}) \cdot \hat{y} + (\boldsymbol{m_r} \times \boldsymbol{m}_{\boldsymbol{r}+\hat{y}}) \cdot (-\hat{x}) \right] - h \sum_{\boldsymbol{r}} m_{\boldsymbol{r}}^z, \tag{S8.1}$$

$$H_{sd} = J_{sd} \sum_{\boldsymbol{r}} s_{\boldsymbol{r}} m_{\boldsymbol{r}}^z, \tag{S8.2}$$

with the ferromagnetic interaction J, the bulk Dzyaloshinskii-Moriya interaction (DMI) D_{bulk}, and DMI due to interface D_{int}. The last term of H_0 represents the Zeeman effect with the external magnetic field h. The unit vector $\hat{e}$ $(= \hat{x}, \hat{y})$ spans the two-dimensional square lattice. Hamiltonian H_{sd} is the sd-type exchange interaction between the conduction electron spin $s_{\boldsymbol{r}}$ in the spin Hall system and the magnetization in CMI. Note that the effects by J_{sd} and D_{int} are suppressed as $\sim 1/d_{\text{CMI}}$ [27], where d_{CMI} is the thickness of CMI.

We study the skyrmion creation by numerically solving the Landau-Lifshitz-Gilbert (LLG) equation,

$$\frac{\partial \boldsymbol{m_r}}{\partial t} = -\frac{\partial H}{\partial \boldsymbol{m_r}} \times \boldsymbol{m_r} + \alpha_{\text{G}} \boldsymbol{m_r} \times \frac{\partial \boldsymbol{m_r}}{\partial t}, \tag{S8.3}$$

where α_{G} is the Gilbert damping constant. We apply the parameter set $\{J = 1, J_{sd} = 0.4, D_{\text{bulk}} = 0.2, D_{\text{int}} = 0.02, h = 0.03, \alpha_{\text{G}} = 0.05\}$, and free boundary condition for the numerical simulation. The unit of time is $\tau_0 \sim 0.7$ ps for $J \sim 10^{-3}$ eV. The typical length scale of the magnetic winding texture is determined by $(J/D) \times a$ with the magnitude of total DMI D. Suppose $a \sim 25$ Å, the skyrmion size shown in Fig. 4 will be ~ 50 nm at most. The movie of the micromagnetic simulation for the skyrmion creation is shown in SI Movie 1, whose snapshots are shown in Fig. 4 in the main text.

SI Movie 1 legend

Movie of the micromagnetic simulation for the skyrmion creation with the parameters of $J = 1$, $J_{sd} = 0.4$, $D_{\text{bulk}} = 0.2$, $D_{\text{int}} = 0.02$, $h = 0.03$, $\alpha_{\text{G}} = 0.05$ and the system size is 401×201.

IX. ELECTRIC FIELD DISTRIBUTION

We here discuss the spatial profile of the electric field. Figure S4 shows the profiles of the electric current and the electric field obtained from the FEM calculation. The electric current profile shown in Fig. S4 (a) is the same as Fig. 3 (c) in the main text. The Ohmic current is obtained from the electric field distribution as $\boldsymbol{j}_e^{\text{Ohm}} = \sigma'_e \boldsymbol{E}$. Near the cavity boundary, the difference between the Ohmic current and the electric current profile (Fig. S4 (a)) is seen, due to the vorticity, which is equivalent to the spin accumulation near the boundary.

[1] E. Saitoh, M. Ueda, H. Miyajima, and G. Tatara, Conversion of spin current into charge current at room temperature: Inverse spin-Hall effect, Appl. Phys. Lett. **88**, 182509 (2006).
[2] S. O. Valenzuela and M. Tinkham, Direct electronic measurement of the spin Hall effect, Nature **442**, 176 (2006).
[3] T. Kimura, Y. Otani, T. Sato, S. Takahashi, and S. Maekawa, Room-Temperature Reversible Spin Hall Effect, Phys. Rev. Lett. **98**, 156601 (2007).

[4] M. I. Dyakonov, Magnetoresistance due to Edge Spin Accumulation, Phys. Rev. Lett. **99**, 126601 (2007).
[5] Y.-T. Chen, S. Takahashi, H. Nakayama, M. Althammer, S. T. B. Goennenwein, E. Saitoh, and G. E. W. Bauer, Theory of spin Hall magnetoresistance, Phys. Rev. B **87**, 144411 (2013).
[6] P. M. Haney, H.-W. Lee, K.-J. Lee, A. Manchon, and M. D. Stiles, Current induced torques and interfacial spin-orbit coupling: Semiclassical modeling, Phys. Rev. B **87**, 174411 (2013).
[7] M. Althammer, S. Meyer, H. Nakayama, M. Schreier, S. Altmannshofer, M. Weiler, H. Huebl, S. Geprägs, M. Opel, R. Gross, D. Meier, C. Klewe, T. Kuschel, J.-M. Schmalhorst, G. Reiss, L. Shen, A. Gupta, Y.-T. Chen, G. E. W. Bauer, E. Saitoh, and S. T. B. Goennenwein, Quantitative study of the spin Hall magnetoresistance in ferromagnetic insulator/normal metal hybrids, Phys. Rev. B **87**, 224401 (2013).
[8] J.-G. Choi, J. W. Lee, and B.-G. Park, Spin Hall magnetoresistance in heavy-metal/metallic-ferromagnet multilayer structures, Phys. Rev. B **96**, 174412 (2017).
[9] M. Matsuo, J. Ieda, K. Harii, E. Saitoh, and S. Maekawa, Mechanical generation of spin current by spin-rotation coupling, Phys. Rev. B **87**, 180402(R) (2013).
[10] R. Takahashi, M. Matsuo, M. Ono, K. Harii, H. Chudo, S. Okayasu, J. Ieda, S. Takahashi, S. Maekawa, and E. Saitoh, Spin hydrodynamic generation, Nat. Phys. **12**, 52 (2016).
[11] D. Kobayashi, T. Yoshikawa, M. Matsuo, R. Iguchi, S. Maekawa, E. Saitoh, and Y. Nozaki, Spin Current Generation Using a Surface Acoustic Wave Generated via Spin-Rotation Coupling, Phys. Rev. Lett. **119**, 077202 (2017).
[12] M. Matsuo, Y. Ohnuma, and S. Maekawa, Theory of spin hydrodynamic generation, Phys. Rev. B **96**, 020401 (2017).
[13] G. Tatara, Spin correlation function theory of spin-charge conversion effects, Phys. Rev. B **98**, 174422 (2018).
[14] H. Tabaei Kazerooni, A. Thieme, J. Schumacher, and C. Cierpka, Electron Spin-Vorticity Coupling in Pipe Flows at Low and High Reynolds Number, Phys. Rev. Applied **14**, 014002 (2020).
[15] R. Takahashi, H. Chudo, M. Matsuo, K. Harii, Y. Ohnuma, S. Maekawa, and E. Saitoh, Giant spin hydrodynamic generation in laminar flow, Nat Commun **11**, 3009 (2020).
[16] Y. Kurimune, M. Matsuo, and Y. Nozaki, Observation of Gyromagnetic Spin Wave Resonance in NiFe Films, Phys. Rev. Lett. **124**, 217205 (2020).
[17] H. Tabaei Kazerooni, G. Zinchenko, J. Schumacher, and C. Cierpka, Electrical voltage by electron spin-vorticity coupling in laminar ducts, Phys. Rev. Fluids **6**, 043703 (2021).
[18] G. Tatara, Hydrodynamic theory of vorticity-induced spin transport, Phys. Rev. B **104**, 184414 (2021).
[19] F. W. Hehl and W.-T. Ni, Inertial effects of a Dirac particle, Phys. Rev. D **42**, 2045 (1990).
[20] C. W. J. Beenakker and H. van Houten, Quantum Transport in Semiconductor Nanostructures, in *Solid State Physics*, Semiconductor Heterostructures and Nanostructures, Vol. 44, edited by H. Ehrenreich and D. Turnbull (Academic Press, 1991) pp. 1–228.
[21] E. I. Kiselev and J. Schmalian, Boundary conditions of viscous electron flow, Phys. Rev. B **99**, 035430 (2019).
[22] O. E. Raichev, Linking boundary conditions for kinetic and hydrodynamic description of fermion gas, Phys. Rev. B **105**, L041301 (2022).
[23] G. Falkovich and L. Levitov, Linking Spatial Distributions of Potential and Current in Viscous Electronics, Phys. Rev. Lett. **119**, 066601 (2017).
[24] I. V. Gornyi and D. G. Polyakov, Two-dimensional electron hydrodynamics in a random array of impenetrable obstacles: Magnetoresistivity, Hall viscosity, and the Landauer dipole, Phys. Rev. B **108**, 165429 (2023).
[25] F. Hecht, New development in FreeFem++, J. Numer. Math. **20**, 251 (2012).
[26] D. A. Papaconstantopoulos, *Handbook of the Band Structure of Elemental Solids* (Springer US, Boston, MA, 2015).
[27] J. Matsuno, N. Ogawa, K. Yasuda, F. Kagawa, W. Koshibae, N. Nagaosa, Y. Tokura, and M. Kawasaki, Interface-driven topological Hall effect in $SrRuO_3$-$SrIrO_3$ bilayer, Sci. Adv. **2**, e1600304 (2016).
